# Case of Worms Discharged through an Opening in the Navel
*We think it right to inform the reader, that this case was communicated to us while Part III. of this volume was in the press, and, of course, before the author had seen the account, inserted in page 251, of another curious case of the same kind.—Editor.


**Published:** 1786

**Authors:** Robert Hamilton

**Affiliations:** Physician at Ipswich


### II. *Case* of Worms discharged through an Opening in the Navel. Communicated in a Letter to* Samuel Foart Simmons, *M. D. F. R. S. by* Robert Hamilton, *M. D. Physician at Ipswich.*

MR. Secamp, an ingenious apothecary, of this place, mentioned to me, some time ago, a case that had lately fallen under the care of Mr. Rudland, surgeon, at Walton, a village about ten miles from Ipswich, near Landguard Fort. It appeared from his account of it to be so extraordinary, that being a few days after in the neighbourhood of Walton, I went thither for the sake of obtaining more satisfactory information on the subject from Mr. Rudland himself. As the patient lived in the village, we visited him together, and the truth of the relation was then fully confirmed to me. — The case is as follows:

A male child, named Parish, a year and a half old, was suspected for several weeks, from

* We think it right to inform the reader, that this case was communicated to us while Part III. of this volume was in the press, and, of course, before the author had seen the account, inserted in page 251, of another curious case of the same kind. — EDITOR.

symptoms

ſymptoms the mother thought ſhe obſerved, to be affected with worms: in other reſpects he was healthy. The navel was protruded about an inch, and appeared inflamed. I took notice of this circumſtance, and queſtioned the mother concerning it. She told me, that the perſon who had the care of the child for a few days after its birth, drew away the bandages that were round the navel too ſuddenly, and with them the remains of the funis, before it had been completely ſeparated: and ſhe added, that though the part healed, it had always appeared tender. To prevent its protruding too much, a bandage had been applied pretty tight over it. Soon after the time the child had been ſuſpected to have worms, on untying this bandage, ſhe obſerved a worm about ſeven inches long, and of the teres kind, according to her deſcription, crawling over the external abdomen. At this ſhe was alarmed, and called in ſome neighbours to ſee it.

In the middle of the navel were two ſmall holes, out of one of which the worm had juſt iſſued. This made her obſerve the child more attentively through the day, and, before night, two more came away at the ſame openings; one of which, on her examining, had protruded itſelf about about two inches, when ſhe pulled it away with her fingers. Next day two more came away in the ſame manner. All theſe worms were from ſix to eight inches in length, and alive when they iſſued out.

Before ſhe applied to Mr. Rudland, ſome of her neighbours had adviſed her to give the child powder of bear's foot, a favourite remedy among the good women here for worms: the quantity, as much at a time as can lay on a ſix-pence. This had been frequently adminiſtered.

No more came away after this for ten days; but, at the end of that time, no leſs than ſix more, as long as the former, found their way out at the ſame openings, and in ſo ſhort a ſpace as twenty-four hours. From this to the time I am receiving this account, no more have appeared, which is about five weeks. The perforations at this time are cloſed; but the navel, which is about the ſize of a walnut, is evidently diſeaſed. This is diſcernible from its colour.

When I made the examination, the abdomen was alſo conſiderably enlarged, which led both Mr. Rudland and myſelf to ſuſpect that more worms were ſoon about to make their exit at the old cicatrices. In this, however, we were miſtaken; miſtaken; for ſeveral months have now elapſed and no more have appeared. The child continues well.

From the time he came under Mr. Rudland's care, tin and other vermifuge medicines had been adminiſtered, but without any appearance of worms in the ſtools. As ſeveral people were called in on the mother's alarm, and were eyewitneſſes of this fact, it can have vouchers enough for its authenticity.

It would appear that the inflammation ſucceeding the raſh pulling away of the remains of the funis, before nature had properly ſeparated it, was followed by adheſions of the neighbouring parts, and that theſe had not only extended to the peritonæum, but to the inteſtine: hence the communication between this canal and the external abdomen at this part.

We find from the hiſtory of caſes, that, from previous inflammation, ſuppuration, and adheſion, communications have been formed between internal cavities and external parts, at which noxious or offenſive ſubſtances have been diſcharged. Stones have made their way from the kidneys in this manner, through muſcles, membranes, and integuments, as we learn from Tulpius (Obſ. l. 4. c. 28.), and alſo from a very curious caſe publiſhed by yourſelf in the Philoſophical Tranſactions, Vol. 68, and have been diſcharged from an abſceſs in the loins. The fæces have alſo eſcaped through the peritonæum, as authors inform us, by means of a like adheſion and ſuppuration, in violent obſtructions of the inteſtines, and have been diſcharged externally, and ſaved the patient's life. In theſe caſes there muſt have been an adheſion of the gut to the peritonæum, otherwiſe they would have fallen into the cavity of the abdomen, and ſpeedily proved fatal.

On the whole, from the caſe above related, we have one inſtance more of the wonderful efforts nature makes to relieve herſelf from things diſturbing the regularity of her operations; and a caution is held out to the unwary nurſe to abſtain from tearing away the remains of the umbilical cord till a proper ſeparation be made, when it will fall off ſpontaneouſly. I have now ſeen two inſtances of the miſchief done by ſuch raſhneſs; nor is the injury trifling to the unfortunate child, which muſt ſuffer from it, more or leſs, while it lives. The navel ſeldom completely heals up; at leaſt it is always tender, and eaſily injured by every little caſualty in purſuing the common affairs of of life. In this caſe, had proper ſtimulating cathartics been applied, it is more than probable they would have been carried through the alimentary canal, and expelled in the common way.

*Ipſwich,*

